# A point prevalence survey and monitoring indicator research on the second batch of national key monitoring and rational use drugs

**DOI:** 10.3389/fphar.2024.1416010

**Published:** 2024-07-05

**Authors:** Li Tang, Hongmei Liu, Shasha Wu, Jing Liu, Xiaoyu Jiang, Yuanyuan Fu, Na Tao, Yong He

**Affiliations:** ^1^ Department of Pharmacy, The Second Affiliated Hospital of Zunyi Medical University, Zunyi, China; ^2^ Department of Pharmacy, Affiliated Hospital of Zunyi Medical University, Zunyi, China

**Keywords:** days of therapy, defined daily dose, National Key Monitoring and Rational Use Drug, monitoring indicator, point prevalence survey, prescribed daily dose

## Abstract

**Background:**

With the remarkable effect of controlling the increase in drug costs by the first batch of National Key Monitoring and Rational Use Drugs (first NKMRUDs), the National Health Commission of the People’s Republic of China releases the second NKMRUDs to further strengthen the reasonable use of drugs. Unfortunately, the second NKMRUDs include some drugs of National Volume-based Procurement and National Essential Medicines, which challenges the management of pharmaceutical affairs on the three kinds of drugs.

**Objective:**

The main objective of this study was to investigate the prevalence of the second NKMRUDs and explore their monitoring indicators.

**Methods:**

An adapted WHO methodology for point prevalence surveys was conducted for the second NKMRUDs. For the monitoring indicators, we sought to explore whether the defined daily dose (DDD) and days of therapy (DOT) can be suitable for the second NKMRUDs through comparing differences between DDD and DOT with the prescribed daily dose (PDD).

**Results:**

Among the 935 included patients, 29.20% of the patients received at least one of the second NKMRUDs. A total of 273 patients were administered with 487 times of the second NKMRUDs. Among them, 162 , 62 , and 49 patients were receiving one, two, and three or more agents, respectively. The most commonly prescribed second NKMRUDs were compound amino acids, budesonide, and ceftazidime. The total DDDs and DOTs of the second NKMRUDs were 3360.68 and 1819.80, respectively, with the PDDs of 1865.26. The deviations (80.17%) of DDDs from PDDs were significantly greater than those (−2.44%) of DOTs.

**Conclusion:**

The prevalence of the second NKMRUDs was obtained by using the adapted PPS methodology at a tertiary university hospital. The DOT indicator is found to more accurately reflect actual consumption than the DDD indicator for second NKMRUDs. It is recommended to use the DOT indicator to monitor second NKMRUDs.

## Introduction

Increasing drug costs is a worldwide challenge (IQVIA institute report, 2023). With the aims of improving the rational usage of medicine and removing perverse economic incentives, China initiates a series of healthcare reforms including the National Essential Medicine (NEM) policy, the National Volume-Based Procurement (NVBP) policy, and National Key Monitoring and Rational Use Drug (NKMRUD) policy (Mao et al., 2022). NEMs are crucial to promoting people health and achieving sustainable development, owing to the importance of “access to safe, effective, quality and affordable” attributes (Liu et al., 2023). The NVBP policy mainly aims to enhance access affordable medications and reduce drug prices through “volume purchase, volume-price linkage, and volume-for-price exchange” (Chen et al., 2021). Meanwhile, NKMRUD policies are released to control the unreasonable increase and standardize the clinical application management in the catalog.

Since significant achievements have been realized after the first batch of NKMRUDs (first NKMRUDs) (Wen et al., 2023), the National Health Commission of the People’s Republic of China releases the list of second batch of NKMRUDs to further strengthen the reasonable use of drugs. Surprisingly, the second NKMRUD list has new features such as antimicrobials and proton pump inhibitors (PPIs), which also belong to NEMs or drugs of NVBP or Provincial Volume-Based Procurement (PVBP). For NEMs, the proportion and sales amount in the tertiary general hospitals should not be less than 30% and 20%, respectively. For the drugs of NVBP, hospitals are forced and encouraged to prescribe them through *Assessment Indicators for Designated Medical Institutions* including compulsory completion of the agreed purchase quantity and rewards for retaining surplus medical insurance funds. Paradoxically, if the second NKMRUDs are strictly implemented restriction on their usage or even exclusion from the hospitals’ regular procurement list like the first NKMRUDs, it will inevitably contradict the favorable incentives provided by NEMs and NVBP or PVBP policies. Therefore, the management departments of pharmaceutical affairs urgently force to find the solution to resolve these contradictions.

A point prevalence survey (PPS) has been widely used to investigate the prevalence of healthcare-associated infections and antibiotic usage since continuous data collection on antibiotic prescription is not possible, owing to the results of the high workload and level of resources required for regular monitoring (Llata et al., 2009; Magill et al., 2014; Versporten et al., 2018). To solve antimicrobial resistance and investigate inappropriate use information on antibiotics, WHO launches *WHO Methodology for Point Prevalence Survey on Antibiotic Use in Hospitals, version 1.1* (WHO PPS methodology) in 2018 (WHO, 2018). The WHO PPS methodology is widely utilized to estimate the antimicrobial usage in various hospitals (Vandael et al., 2020; Khursheed et al., 2023). As the most used indicators for antimicrobial stewardship programs (ASPs), defined daily dose (DDD) or days of therapy (DOTs) are selected as quantitative monitoring indicators in different countries (European Centre for Disease Prevention and Control, 2022 and Centers for Disease Control and Prevention, 2024).

As the WHO PPS methodology can be adapted and tailored for specific purposes, we were inspired to investigate the prevalence of second NKMRUDs in a tertiary university hospital in Guizhou province, China. Meanwhile, we sought to explore whether DDD or DOT could be used as the indicators for second NKMRUDs, as compared with the prescribed daily dose (PDD), which reflected the actual average dose of the prescriptions. Importantly, we tried to find the possibility of the solution for the contradiction among second NKMRUDs, NEMs, and NVBP or PVBP policies.

## Methods

### Setting and study design

A WHO PPS methodology for second NKMRUDs was conducted at the Second Affiliated Hospital of Zunyi Medical University (second AHZMU) in Guizhou Province, China, for three consecutive weekdays in July 2023. The second AHZMU is a 959-bed, tertiary A-level comprehensive university hospital. The annual number of hospital discharges reaches 42,969, and the annual number of surgeries is 13,494 (data updated until 16 January 2024). A total of 48 specialties are set including orthopedic, ophthalmology, neurology, and other provincial priority clinical specialties.

We utilized a modified WHO PPS methodology according to the study objectives and the characteristics of second NKMRUDs. A corresponding excel sheet was designed to collect information on the clinical information about patients through a medical chart.

### Ward selection

The emergency department and psychiatric ward were excluded in the study. All other acute care inpatient wards at second AHZMU were included in the study.

### Patient selection

We enrolled all patients who were admitted on a study ward before 8 a.m. on the day of the survey, who had not been discharged from the ward by the time of the data collection. Patients were excluded if they were absent from the wards at the time of the survey.

### Data collection and definitions

Prior to conducting PPS, the data collection team received data collection and methodology training to ensure data entry standardization. Two team members formed study pairs each day, alternating every half day.

Patient-level data including age, gender, medical specialty, renal function, primary diagnosis, comorbidities, and detailed information on second NKMRUDs (drug, dosage, route, duration, and indication) were collected. All data were available through the Hospital Information System. Wards were divided into adult medical wards, adult surgical wards, oncology wards, pediatric ward, neonatal ward, mixed ward, and intensive care ward.

### Indicators of calculating drug consumption

DDDs and DOTs were collected as the indicators for drug consumption. For DDD calculation, it was obtained by dividing the total drug consumption of each drug into its corresponding DDD value, which is available online on the website of Anatomical Therapeutic Chemical Classification System and the DDD index. For DOT calculation, one DOT represents the administration of a single drug on a given day, regardless of the number of administered doses or dosage strength. One patient simultaneously receiving two drugs of second NKMRUDs would be considered receiving two DOTs (one for each drug administered) and so on, in accordance with the number of drugs of second NKMRUDs received daily (Polk et al., 2007).

In order to judge whether DDDs or DOTs is better to be selected as the indicators for second NKMRUDs, PDDs were chosen as the reference indicator. For PDD calculation, it was obtained through dividing the total utilization of each drug into its calculated PDD, which was defined as reflecting the usually prescribed dose in adult hospitalized patients with normal renal function (de With et al., 2006).

Deviations between DDDs and DOTs from PDDs were calculated through DDDs minus PDDs and DOTs minus PDDs, respectively. The deviation ratios of DDDs and DOTs were calculated using the following formulas, respectively:

$$Deviation\;ratio\;of\;DDDs\;and\;PDDs=\frac{\left(DDDs-PDDs\right)}{PDDs}\times 100\%,$$

$$Deviation\;ratio\;of\;DOTs\;and\;PDDs=\frac{\left(DOTs-PDDs\right)}{PDDs}\times 100\%.$$

### Statistical analysis

Data analysis was performed using IBM SPSS 26 software. Descriptive data were described as the number (percentage). The Wilcoxon rank-sum test was used for non-normally distributed variables. A *p*-value of less than 0.05 was considered statistically significant.

### Ethical considerations

Ethical approval (KYLL-2023-056) was proved by the Ethics Committee of second AHZMU, waiving the requirement for individual consent.

## Result

### Patient baseline characteristics

There were 1,044 eligible patients who were on the ward when the assessment was conducted at 8 a.m. prior to the survey. In total, 109 patients were excluded since they planned to be discharged or were off the wards at the time of the survey. Finally, 935 patients were included for the following analysis.

The majority of the wards of the included patients were the oncology wards (n = 350), adult surgical wards (n = 315), adult medical wards (n = 212), pediatric wards (n = 32), intensive care wards (n = 12), mixed wards (n = 10), and neonatal medical wards (n = 4). The median age of the patients was 55 (IQR 43-66) years, and 481 (51.44%) were male patients. The median hospital length of stay before conducting the PPS was 5 days (IQR 2–14 days). Further clinical and demographic characteristics of patients are listed in Table 1.

**TABLE 1:** Characteristics of patients using second NKMRUDs included in the survey.

	All patients	Using second NKMRUD
Patients, n	935	273
Male, n (%)	481 (51.44%)	171 (62.64%)
Age, n (%)		
Neonate	8 (0.86%)	0
0–14	74 (7.91%)	30 (10.99%)
15–29	48 (5.13%)	12 (4.40%)
30–44	111 (11.87%)	13 (4.67%)
45–59	360 (38.50%)	86 (31.50%)
60+	334 (35.72%)	132 (48.35%)
Ward, n (%)		
Pediatric wards	32 (3.42%)	21 (7.69%)
Neonatal wards	4 (0.43%)	0
Adult medical wards	212 (22.67%)	122 (44.69%)
Adult surgical wards	315 (33.69%)	76 (27.84%)
Oncology wards^#^	350 (37.43%)	38 (13.92%)
Intensive care wards	12 (1.28%)	12 (4.40%)
Mixed wards	10 (1.07%)	4 (1.47%)
Length of stay in days until PPS is performed, median (IQR)	5 (2–14)	6 (3–11)

^#^The oncology wards include three specialties: head and neck oncology wards, thoracic oncology wards, and abdominal oncology wards.

### Prevalence and characteristics of second NKMRUD use

The prevalence and percentage of second NKMRUD prescription are shown in Table 2. Overall, 29.20% (273/935) of patients were receiving at least one second NKMRUD during the time of the survey. Among hospital wards, the prevalence of patients using second NKMRUD ranged from 0 in the neonatal ward to 100% in the intensive care ward. A total of 273 patients were administered 487 times of second NKMRUDs. In total, 162 patients were receiving one agent. A total of 62 patients were receiving two agents, and 49 patients were receiving three or more agents.

**TABLE 2:** Prevalence rates of second NKMRUDs among the wards.

Ward	Patients using second NKMRUDs		Second NKMRUD prescription	
	Number	Prevalence^#^	Number	Percentage
Total	273	29.20%	487	100%
Pediatric wards	21	65.63%	25	5.13%
Neonatal wards	0	0	0	0
Adult medical wards	122	57.55%	211	43.33%
Adult surgical wards	76	24.13%	132	27.10%
Oncology wards	38	10.86%	53	10.88%
Intensive care wards	12	100%	58	11.91%
Mixed wards	4	40%	8	1.64%

^#^Prevalence was determined by dividing the number of patients using second NKMRUDs by the number of patients in total and the corresponding ward, as listed in Table 1.

The most commonly prescribed second NKMRUDs were compound amino acids, budesonide, and ceftazidime, as listed in Table 3. The overwhelming majority of all second NKMRUD prescriptions was parenteral formulation (83.57%, n = 407). The oral formulation of second NKMRUDs constituted significantly fewer patients (1.23%, n = 6). For the indications of the second NKMRUD use, 278 (57.08%) of all the prescriptions were administered for therapeutic purpose, 182 (37.37%) for adjuvant therapy, and 27 (5.54%) for prophylaxis. Meanwhile, the median extending length of time for the second NKMRUDs was 5 days (IQR 2–8 days), which was calculated from the days of conducting PPS backward to the days of the beginning of second NKMRUD prescription.

**TABLE 3:** Patient-level information about second NKMRUDs.

Routes of administration, n (%)	
Parenteral	407 (83.57%)
Oral	6 (1.23%)
Inhalational	74 (15.20%)
Types of indications, n (%)	
Therapeutic	278 (57.08%)
Adjunctive	182 (37.37%)
Preventative	27 (5.54%)
Reason in notes, n (%)	
YES	421 (86.45%)
NO	66 (13.55%)
Number of second NKMRUDs, n (%)	
1	162 (59.34%)
2	62 (22.71%)
3	24 (8.79%)
4	11 (4.03%)
5	2 (0.73%)
6	9 (3.30%)
7	3 (1.10%)
Top five prescriptions for second NKMRUDs, n (%)	
Compound amino acid	95 (19.51%)
Budesonide	74 (15.20%)
Ceftazidime	40 (8.21%)
Omeprazole	29 (5.95%)
Human albumin	27 (5.54%)
Pantoprazole	27 (5.54%)
Drugs of NVBP or PVBP, n (%)	281 (57.70%)
NEMs, n (%)	183 (37.58%)
Length of second NKMRUD use in days, median (IQR)^#^	5 (2–8)

^#^Include the day of the survey if the second NKMRUDs were used on the day of the survey.

### Comparison between DDDs and DOTs with PDDs

The detailed information on the DDD values; calculated PDD values; and the policy attributes including NVBP, PVBP, NEM, and National Health Insurance Drugs is shown in Table 4. Importantly, the proportion of NVBP or PVBP medicines was 48%, and the proportion of NEMs was 24%.

**TABLE 4:** Characteristics of the second NKMRUDs in our hospital.

Drug	Category	ATC code	Number of specifications	DDD	PDD	Adm.R	Drug of NVBP	Drug of PVBP	NEMs	National Health Insurance drug
Cefoperazone + sulbactam	ASU	J01DD62	2	4 g	4.31 g	P	NO	NO	NO	B
Cefotaxime	ASU	J01DD01	1	4 g	4.91 g	P	NO	YES	NO	NO
Meropenem	ASU	J01DH02	2	3 g	2.78 g	P	YES	NO	NO	NO
Levofloxacin	ASU	J01MA12	2	0.5 g	0.44 g	P, O	YES	NO	NO	NO
Piperacillin + tazobactam	ASU	J01CR05	2	14 g	9.09 g	P	YES	YES	YES^e^	NO
Ceftazidime	ASU	J01DD02	1	4 g	4.14 g	P	YES	NO	YES	NO
Omeprazole	ATM	A02BC01	2	20 mg	79.47 mg	P, O	YES^a^	YES^d^	YES	NO
Pantoprazole	ATM	A02BC02	2	40 mg	88.77 mg	P, O	YES^b^	YES^a^	NO	B^a^, A^b^
Esomeprazole	ATM	A02BC05	2	30 mg	103.37 mg	P, O	NO	YES	NO	B^d^
Lansoprazole	ATM	A02BC03	1	30 mg	51.25 mg	P	YES	NO	NO	NO
Famotidine	ATM	A02BA03	1	40 mg	40 mg	P	NO	NO	YES	NO
Papaverine	ATM	A03AD01	1	0.1 g	0.06 g	P	NO	YES	NO	NO
Betahistine	NS	N07CA01	2	24 mg	22.19 mg	P, O	NO	NO	NO	B
Nicotinic acid	CS	C04AC01	1	0.2 g	0.55 g	P	NO	NO	NO	NO
Alprostadil	CS	G04BE01	1	20 μg	16.67 μg	P	NO	NO	NO	NO
Budesonide	RS	R03BA02	2	1.5 mg	2.83 mg	INH	YES^c^	NO	YES	NO
Oxiracetam	NS	N06BX07	3	NONE	NONE	P, O	NO	NO	NO	C
Monosialotetrahexosylganglioside	NS	N07XA	1	NONE	NONE	P	NO	NO	NO	C
Dezocine	NS	N02AX03	1	NONE	NONE	P	NO	NO	NO	NO
Edaravone	NS	N07XX14	1	NONE	NONE	P	NO	YES	NO	B
Human albumin	BBFO	B05AA01	1	NONE	NONE	P	NO	NO	NO	NO
*Ginkgo biloba* extract	NONE	NONE	1	NONE	NONE	P	NO	NO	NO	B
Aceglutamide	NONE	NONE	2	NONE	NONE	P	NO	NO	NO	B
Fosfocreatine	NONE	NONE	1	NONE	NONE	P	NO	NO	NO	C
Compound amino acid	NONE	NONE	10	NONE	NONE	P	NO	NO	YES^f^	A^g^, B^h^

ASU, anti-infective for systemic use; ATM, alimentary tract and metabolism; NS, nervous system; CS, cardiovascular system; RS, respiratory system; BBFO, blood and blood-forming organs; P, parenteral; O, oral; INH, inhalational; NVBP, National Volume-Based Procurement; PVBP, Provincial Volume-Based Procurement; NEMs, National Essential Medicines; A, Class A Medicare drugs; B, Class B Medicare drugs; C, Class C Medicare drugs; ^a^, for injection; ^b^, for enteric-coated tablet; ^c^, for suspension; ^d^, for enteric capsule; ^e^, for 2.25 g specification; ^f^, for 18AA-II; ^g^, for 18AA-I/18AA-II//18AA-V; ^h^, For 18AA-V-SF/9AA.

The total number of second NKMRUDs prescribed at the second AHZMU was 25, in which 16 kinds of drugs were assigned with the DDD value by the ATC/DDD system. The total PDDs, DDDs, and DOTs of the second NKMRUDs were 1865.26, 3360.68, and 1819.80, respectively (Table 5). For total second NKMRUDs, the deviations (80.17%) of DDDs from PDDs were significantly greater than those (−2.44%) of DOTs from PDDs (*p* < 0.05). The top three of the largest absolute deviations of DDDs from PDDs were omeprazole (297.36%), esomeprazole (244.57%), and nicotinic acid (175.03%). The top three of the slight absolute deviation of DOTs from PDDs were cefotaxime (31.16%), alprostadil (27.19%), and levofloxacin (−18.87%).

**TABLE 5:** Comparison between DDDs and DOTs with PDDs.

Category	PDDs	DDDs#		DOTs	
		*n*	Deviation from PDDs	*n*	Deviation from PDDs
Total	1865.26	3360.68	1495.42 (80.17%)	1819.80	−45.46 (−2.44%)
Cefoperazone + sulbactam	68.68	74.00	5.32 (7.75%)	60.00	−8.68 (−12.64%)
Cefotaxime	19.06	23.40	4.34 (22.77%)	25.00	5.94 (31.16%)
Meropenem	163.67	151.67	−12.00 (−7.33%)	176.00	12.33 (7.53%)
Levofloxacin	160.23	141.00	−19.23 (−12.00%)	130.00	−30.23 (−18.87%)
Piperacillin + tazobactam	133.17	86.46	−46.71 (−35.08%)	130.00	−3.17 (−2.38%)
Ceftazidime	224.15	232.00	7.85 (3.50%)	228.00	3.85 (1.72%)
Omeprazole	158.95	631.60	472.65 (297.36%)	167.80	8.85 (5.57%)
Pantoprazole	139.69	310.00	170.31 (121.92%)	140.00	−0.31 (−0.22%)
Esomeprazole	205.09	706.67	501.58 (244.57%)	170.00	−35.09 (−17.11%)
Lansoprazole	42.73	73.00	30.27 (70.84%)	43.00	0.27 (0.63%)
Famotidine	42.00	42.00	0.00	43.00	1.00 (2.38%)
Papaverine	8.00	4.80	−3.2 (−40.00%)	8.00	0.00
Betahistine	68.95	63.75	−5.20 (−7.54%)	69.00	0.05 (0.07%)
Nicotinic acid	36.36	100.00	63.64 (175.03%)	37.00	0.64 (1.76%)
Alprostadil	22.80	19.00	−3.80 (−16.67%)	29.00	6.2 (27.19%)
Budesonide	371.73	701.33	329.60 (88.67%)	364.00	−7.73 (−2.08%)

^#^The data on the group of “deviation from DDDs” and group of “deviation from DOTs” were taken in absolute value and statistically analyzed. In addition, there was a significant difference between the absolute values of the data on the two groups, *p* < 0.05.

## Discussion

A WHO PPS methodology was conducted to investigate the prevalence of second NKMRUDs in a tertiary university hospital. The PPS methodology is a practical monitoring tool that provides information on medication use at a specific point in time and a closer approximation of actual medication use than aggregated data (Saleem et al., 2020). The PPS methodology is widely used to investigate the use of antimicrobials in ASPs (Alothman et al., 2020).

In this study, we found that the prevalence of second NKMRUD use was 29.20%. To the best of our knowledge, this is the first time that the PPS methodology is utilized to investigate the use of the second NKMRUDs in hospitals. Interestingly, we found that the same inspired work was conducted to investigate sedation practices in intensive care units (Richards-Belle et al., 2016). We suggest that the prevalence rate is relatively low based on our personal experience. The ward of the highest prevalence was the intensive care ward, in which all the patients had been prescribed with the average of 4.83 kinds of second NKMRUDs. The reason may be due to the complexity and severity of the patients’ complication or comorbidity. Meanwhile, the wards with the lowest prevalence were the neonatal ward and oncology ward. The patients in the adult medical wards with the largest proportion were prescribed with the maximum number of second NKMRUDs. The majority of patients used one or two second NKMRUDs. Compound amino acids, budesonide, and ceftazidime were the top three prescriptions of second NKMRUDs. Importantly, the catalog of the second NKMRUDs consists of NEMs (24%) and drugs of NVBPs or PVBPs (48%) in our hospital.

For first NKMRUDs, the only two reported works focus on the utilization of interrupted time-series analysis to estimate the impact of first NKMRUDs (Li et al., 2022; Wen et al., 2023). The implementation of first NKMRUDs effectively reduces their expenses. Furthermore, some hospitals have implemented elimination and restriction measures for first NKMRUDs. Unfortunately, since the attributes of intent to meet the priority healthcare needs of a population for NEMs (Persaud et al., 2019) and improved the medication affordability attributes for NVBPs (Yuan et al., 2021), implementation of second NKMRUDs has to face a conflict of restriction usage and encouragement usage. Therefore, it is essential to select appropriate indicators for monitoring the second NKMRUDs, which can be a reliable practice to resolve the contradiction between the policy on NEMs or PVBP and second NKMRUDs. On one hand, monitoring indicators obviously facilitate the management of pharmaceutical affairs for various types of hospitals. On the other hand, an abnormal increase can be detected in time through the tracking of the monitoring indicators, providing an extraordinary warning.

In this study, we attempt to explore whether DDDs or DOTs could be used as the indicator for second NKMRUDs, as compared with PDDs. In drug utilization monitoring and research, a rough estimation of the PDD could provide a close approximation of drug use (Gagliotti et al., 2014). However, PDD suffers from the complex and time-consuming calculations. Therefore, PDD was always used as the reference. Meanwhile, DDDs and DOTs are the two main indicator measures in the ASPs. DDDs recommended by WHO focus on population-based parameters, assuming that the entities of patients and hospitals are homogenous. DOTs recommended by the Infectious Diseases Society of America/the Society for Healthcare Epidemiology of America guidelines for ASP classify the days of antimicrobial drug usage based on patient-level exposure (Barlam et al., 2016; Ababneh et al., 2021). The crucial advantage of the DDD indicator is that DDDs allow for the standardized comparison of the aggregate antimicrobial usage between hospitals. However, DDDs are always affected by dosage adjustment, especially in the pediatric ward or intensive care ward (Baier et al., 2022; Antunes et al., 2023). On the contrary, the main advantage of DOTs is unaffected by dosage adjustment. However, DOTs will overestimate the use of drugs given in multiple doses per day and more difficult to measure without computerized pharmacy records.

In this study, we found that the total absolute deviations (80.17%) of DDDs from PDDs were much greater than the absolute deviations (−2.44%) of DOTs from PDDs for the second NKMRUDs, indicating that DOTs might be more suitable for monitoring second NKMRUDs. DDDs of PPIs deviated significantly from the PDDs due to large differences between the actual administered dose and its DDD value: omeprazole (DDD = 20 mg, PDD = 79.47 mg), pantoprazole (DDD = 40 mg, PDD = 88.77 mg), lansoprazole (DDD = 30 mg, PDD = 51.25 mg), and esomeprazole (DDD = 30 mg, PDD = 103.37 mg). A slight absolute deviation (−7.86%) of DDDs from PDDs was observed for six antimicrobials. Among the six antimicrobials, cefotaxime (22.77%) and piperacillin–tazobactam (−35.08%) showed relatively large absolute deviations. Previous studies have also found that the PDDs of many antimicrobials do not correspond to the DDDs (Muller et al., 2006; Nunes et al., 2022). With KD et al. found that DDDs overestimated total antimicrobial use by 32% compared to PDDs (de With et al., 2009). The similar overestimation (28%) was also reported by Först et al. (2017). This was inconsistent with our results. The reason may be related to the quite small kinds of antimicrobials in our survey. The following large absolute deviations of DDDs from PDDs were nicotinic acid (175.03%), budesonide (88.67%), and poppycock (−40%). Meanwhile, the deviations of DOTs range from 0% to 31.16%. The DOTs of cefotaxime showed the largest absolute deviation (31.16%) from PDDs, but the similar deviation (22.77%) of DDDs was also observed. The similar trend was also observed for DOT deviation (27.19%) and DDD deviation (−16.67%) for alprostadil. Therefore, the above results indicate that DOTs might be more suitable for monitoring second NKMRUDs than DDDs.

The real goal of the DDD indicator is to estimate the days of actual antimicrobial prescription (Ibrahim and Polk, 2014). However, it is well-known that the DDDs cannot accurately estimate the days when the administered daily dose is significantly different from the DDD value. Meanwhile, the advantages of the DOT indicator are unaffected by the administered daily dose. Therefore, the DOT indicator should always be recommended to monitor the antimicrobial consumption in the intensive care ward or pediatric ward. In this study, we find that the DOT indicator seems to be more suitable for monitoring second NKMRUDs. The DOT indicator can not only recognize the patterns of drug combination and monotherapy but also estimate the actual drug utilization from patient-level prescription data (Momattin et al., 2018; Kallen et al., 2019).

There are some limitations to our study. This study was conducted in a single center with a local prescribing decision, prescribing pattern, and patient mix. In the future work, we attempt to conduct the multi-center study to testify the results. Additionally, the PPS methodology just represents the characterization of second NKMRUD use in the time of survey.

## Conclusion

The prevalence rate, patient, and prescription characteristics of the second NKMRUDs were reported in this study. This is the first time that the PPS methodology is adapted to investigate the baseline information for the management of second NKMRUDs in the hospital. Our results indicate that DOT is more suitable as the indicator for monitoring the second NKMRUDs. To the best of our knowledge, this is also the first time to explore the monitoring indicator for the management of the second NKMRUDs. It is recommended that the DOT indicator should be selected as the monitoring indicator for second NKMRUDs in the hospitals, which may promote the refined management of second NKMRUDs.

## Data Availability

The raw data supporting the conclusions of this article will be made available by the authors, without undue reservation.
